# The Three-Dimensional Morphology of Growing Dendrites

**DOI:** 10.1038/srep11824

**Published:** 2015-07-03

**Authors:** J. W. Gibbs, K. A. Mohan, E. B. Gulsoy, A. J. Shahani, X. Xiao, C. A. Bouman, M. De Graef, P. W. Voorhees

**Affiliations:** 1 Department of Materials Science and Engineering, Northwestern University, Evanston, IL; 2 Department of Electrical and Computer Engineering, Purdue University, West Lafayette, IN; 3 Advanced Photon Source, Argonne National Laboratory, Lemont, IL 60439; 4 Department of Materials Science and Engineering, Carnegie Mellon University, Pittsburgh, PA.

## Abstract

The processes controlling the morphology of dendrites have been of great interest to a wide range of communities, since they are examples of an out-of-equilibrium pattern forming system, there is a clear connection with battery failure processes, and their morphology sets the properties of many metallic alloys. We determine the three-dimensional morphology of free growing metallic dendrites using a novel X-ray tomographic technique that improves the temporal resolution by more than an order of magnitude compared to conventional techniques. These measurements show that the growth morphology of metallic dendrites is surprisingly different from that seen in model systems, the morphology is not self-similar with distance back from the tip, and that this morphology can have an unexpectedly strong influence on solute segregation in castings. These experiments also provide benchmark data that can be used to validate simulations of free dendritic growth.

Dendrites exist almost everywhere, from industrial metal castings to Li metal anodes in Li-ion batteries. Not only are dendrites ubiquitous, they also have a profound effect on the properties of materials; for example, their morphology has a major influence on materials properties as a result of the coupling between the dendrite arms and the chemical non-uniformity of the solid^1^ and in Li-ion batteries, dendritic growth is a major failure mechanism^2^,^3^,^4^. This combination of prevalence and influence on material performance means that the dendrite growth kinetics and morphology have profound technological implications. In addition, dendrites are also an example of an out-of-equilibrium pattern forming system^5^,^6^. Here the focus is on understanding the processes underlying the formation of the complicated dendrite morphology from a featureless liquid.

The most interesting stage of growth is the initial formation of the dendrite in which the dendrite grows into a supersaturated or undercooled liquid, the so-called free-growth phase, since during this stage, the morphology of the dendrite is largely set as the secondary branches start to form along the primary arm. The subsequent slow cooling to 100% solid has a comparatively small, but still important, effect on the dendrite morphology and segregation patterns^7^. Great insights into the dynamics of free-growth stage of dendritic growth have been gained by experiments with metals in thin cells and transparent organic materials^8^,^9^,^10^. However, as a result of the quasi-two-dimensional nature of growth in thin cells, and the limited number of views available in the transparent materials, it has not been possible to measure the three-dimensional (3D) structure of growing dendrites and, in particular, their side-branch morphology. This is particularly unfortunate, since the side-branch morphology is intimately related to the properties of the resulting solid. Furthermore, the similarity between the entropy of fusion of certain transparent organics and metals leads one to believe that the transparent organics are good analogues of growing metallic dendrites^11^, yet this has not been tested due to the inability to observe metallic dendrites growing into a supersaturated or undercooled liquid. An alternative approach is to determine the three-dimensional dendrite morphology by quenching a metallic alloy during growth and then performing a post-quench analysis. However, the quenching process can introduce artifacts in the dendrite morphology. Thus, the three-dimensional morphological development of dendrites during the critically important free-growth stage is completely unstudied. We show through X-ray synchrotron radiation and a novel tomographic reconstruction algorithm that it is possible to image and quantitatively analyze the 3D morphology of growing dendrites during the free-growth stage.

Many theories and simulations exist that address the three-dimensional morphology of growing dendrites^5^,^6^. There are many theoretical predictions of the shape^12^ and branching behavior^13^,^14^ of free-growing dendrites and with improvements in computational speed and algorithms, three-dimensional simulations of dendritic growth are on the verge of being possible^15^. However, there are no three-dimensional morphological datasets that can be compared to these theoretical models and simulations.

X-ray radiation can penetrate metallic samples, and thus provides the information required to determine the 3D morphology of growing dendrites. However, since the dendrites are not static, the structure evolves while the tomographic data are being recorded, leading to inaccuracies in the reconstruction. In past work, this has limited tomographic studies to the later stages of the solidification process, where there is already a well-developed dendritic solid-liquid mixture^16^,^17^; thus, these studies can only measure the evolution of a preexisting dendritic mixture and not the initial dendrite formation. Efforts aimed at reducing the time required to collect a dataset have resulted in scan times of as little as 0.15 seconds^18^; however, this temporal resolution comes at the expense of spatial resolution and the resulting data are too coarsely resolved to make out the details of the dendritic morphology. Therefore, there is a need for a measurement technique with both spatial and temporal resolution that are similar to the size and time scales at which the dendrites are growing into the supersaturated liquid that will also allow for 3D characterization of the dendrites.

We achieve the necessary spatial and temporal resolution improvements by using the time-interlaced model-based iterative reconstruction (TIMBIR) methodology see supplementary information and Refs. 19,20. This was done at the 2-BM beamline at Argonne National Laboratory’s Advanced Photon Source. The spatial and temporal discretization sizes are 0.65 μm voxels (edge length) and 1.8 seconds between each 3D reconstruction. We characterize dendritic growth in an Al-24wt%Cu alloy, which is an ideal system for this experiment because it is well characterized and the relatively high copper content provides good contrast between the solid and liquid due to the large difference in atomic number between aluminum and copper and the different compositions between the solid and liquid.

The experiment uses a 1 mm diameter sample that is cooled at a rate of 2 °C per minute. Given the size of the sample and the cooling rate, the temperature is uniform within the sample, and thus the dendrites grow into a supersaturated liquid and the growth is by solute diffusion. Because the dendrite growth dynamics during the free-growth stage are diffusion limited, the cooling rate does not have a critical influence on the dendrite morphology or growth behavior.

## Results

**Figure 1:**
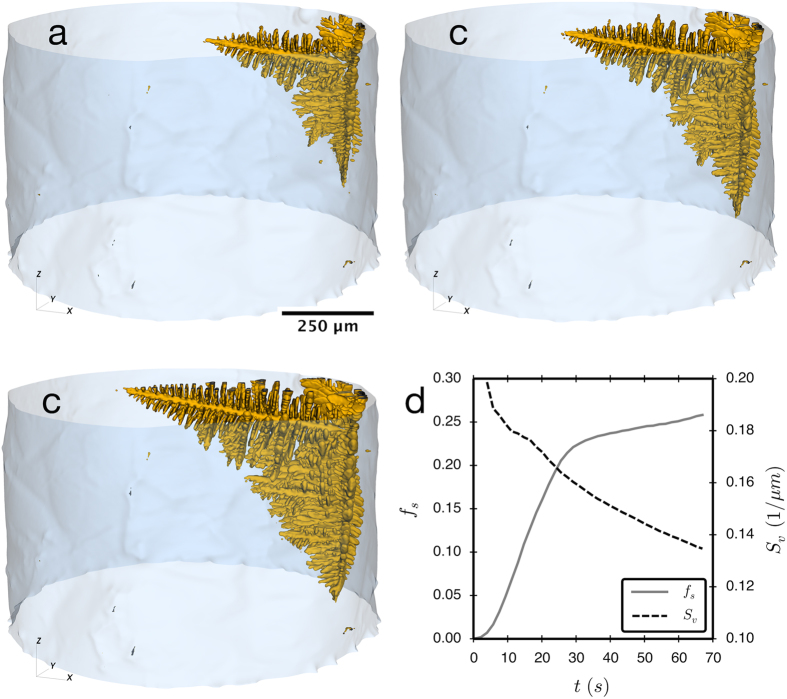
Morphology of the full sample at (**a**) 5.8, (**b**) 7.6 and (**c**) 9.4 seconds after the start of solidification. The dendrite that is growing out into the sample is the one that is isolated and analyzed. Solid fraction (*f*_*s*_) and interfacial area per unit volume (*S*_*v*_) are shown in (**d**).

The evolution of the sample during solidification at a few times is shown in Fig. 1. A single dendrite forms on the side of the sample with primary arms growing out across the sample and four arms growing near the sample walls. By symmetry, all of these arms are growing in the <100> direction, as are the secondary and tertiary arms. The evolution of the volume fraction of the solid phase (*f*_*s*_), shown in Fig. 1d, indicates that the sample is significantly undercooled when nucleation occurred, leading to a rapid rise in the solid fraction to just over 20% solid by volume. At this point, the supersaturation is exhausted and further solidification is driven by the cooling rate of the furnace. The datasets that are analyzed in this work are in the solute supersaturation-driven growth regime, which closely matches with theoretical and simulation work on dendrite evolution behavior.

The surface area per unit volume of solid phase (*S*_*v*_) is also shown in Fig. 1d. This quantity is inversely proportional to the average feature size in the system; thus, the decreasing *S*_*v*_ is indicative of the average feature size increasing. The decrease, however, is only about 30% of the value after 5 seconds of solidification. In comparison the volume fraction of solid has increased by nearly a factor of 2.5. So, while the solid-liquid interfacial area increases dramatically during solidification, the volume fraction increases as well leading to a small change in *S*_*v*_. It is interesting to note that the slope of *S*_*v*_ does not seem to be affected by this change from undercooling-driven solidification to cooling-rate-driven solidification that occurs at around 30 seconds and 20% solid volume. Since the change in the volume fraction of solid is relatively small in the later slow-cooling period, the decrease in *S*_*v*_ is largely a result of a decreasing rate of total interfacial area creation that results from the increasing importance of coarsening as the transformation proceeds.

A nearly free-growing dendrite is isolated from the ensemble of growing dendrites, and analyzed both as a function of time and as a function of distance from the dendrite tip. The term “nearly free-growing” is employed to indicate that at the early stages of growth the side branches are not interacting with surrounding dendrites and that the dendrite is growing into a liquid at a supersaturation that is fixed far from the dendrite tip. The dendrite is growing at a constant speed of approximately 69 μm/s.

Each reconstructed frame uses data that is a 1.8 second window, so the reconstruction at each time must contain a nominal temporal blurring of at least 1.8 seconds. Because the dendrite tip is growing at a relatively high speed compared to this amount of blurring, the interface morphology at the dendrite tip cannot be determined with confidence; however, the slower moving side branches behind the tip are easily resolved, as is the average location of the tip, which is used to calculate the tip velocity. The tip radius can be estimated using the marginal stability criterion^21^ with literature values of capillary length^22^, diffusion coefficient^23^, and the measured growth velocity. This results in a calculated tip radius of approximately 1.5 μm, which is consistent with the thickness of the primary dendrite arm.

The morphology of the dendrite is characterized by discretizing the interface into many small patches and calculating the principal curvatures, *κ*_1_ and *κ*_2_, of every patch. Using this information, the interface shape distribution (ISD) can be determined^24^. In this case, the ISD is the probability of finding a patch of interface with a given shape factor, *S* = 2/*π*tan((*κ*_2_ + *κ*_1_)/(*κ*_2_ − *κ*_1_)) and curvature 
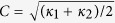
, where *S* = ±1, *S* = ±0.5, or *S* = 0 when a patch of interface is spherical, cylindrical, or hyperbolic respectively^25^. The sign of *S* indicates whether the patch is concave towards the liquid (*S* < 0) or solid (*S* > 0). The curvature indicates the curvedness of the interfacial patch, with *C* = 0 being a flat interface.

**Figure 2:**
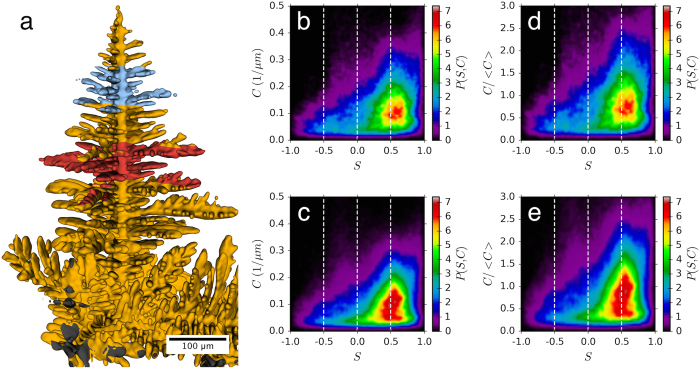
Interfacial shape distributions for two 75 μm thick slices normal to the growth direction of the nearly free-growing dendrite at 9.0 seconds after nucleation. The blue section is centered at 125 μm from the dendrite tip and corresponds to the ISDs in 2b and 2d; the red section is centered at 250 μm from the tip and corresponds to the ISDs 2c and 2e. The ISDs in 2d and 2e have their vertical axes scaled by the average curvature, 〈*C*〉.

The ISDs in Fig. 2 show that the dendrite is composed of predominantly solid cylindrical patches with spherical caps; these represent the secondary and tertiary dendrite arms. The hyperbolic or saddle-shaped patches are mostly found in the transition between the main dendrite arm and the secondary arms. The liquid-cylindrically shaped patches are mainly on the primary stem of the dendrite, between the secondary arms. Comparing two ISDs that were calculated for different positions along the dendrite reveals that the single peak in the data from the region that is closer to the dendrite tip (Fig. 2b) elongates along the *S* = 0 line in Fig. 2c. This is indicative of a transition from many secondary dendrite arms with very similar curvatures to a combination of larger secondary arms and smaller tertiary arms. A particularly surprising result is that there is a considerable amount of interfacial area that is nearly flat, note the substantial region of nonzero probability in the region *C* < 0.05. Because *S* is difficult to determine in experimentally measured structures with nearly flat interfaces, nearly flat interfaces yield a band of probability over a wide range of *S* for low *C*, exactly as seen in Fig. 2. In this case, this band of probability at low *C* is a result of the nearly flat interfaces of secondary dendrite arms. Such nearly flat interfaces do not exist in transparent metal analogues.

In addition, there is a general elongation of the ISD in the *C* direction for any *S* on going from Fig. 2b,c. One possible explanation for this elongation is that it results from a change in the average value of *C*, or characteristic length scale (inverse of *C*), with distance back from the tip. To determine if this is the case, we plot the ISD’s as function of *C*/〈*C*〉 in Fig. 2d,e, where 〈*C*〉 is the surface integral of *C* divided by the total surface area. If the two scaled ISD’s are identical then the morphology is self-similar and would suggest that the dendrite morphology self-organizes into a unique morphology. Thus, the morphology of a dendrite could be described at any time by the ISD as a function of *C*/〈*C*〉, and the value of 〈*C*〉. However, the ISD’s are not self similar, see Fig. 2d,e. For example, the peaks of the ISD are clearly different with ISD taken further back from the tip having a peak that is much more elongated along *S* = 0.5. There is also a flattening of the secondary arms with increasing distance from the tip, as shown by the increase in relative area of interfaces around *C*/〈*C*〉 < 0.5 in Fig. 2e compared to Fig. 2d. This lack of self-similarity indicates that the different dynamics associated with the secondary and tertiary dendrite growth does not simply scale with the average size scale of the structure. These ISD’s also provide the experimental data needed to compare with future simulations of dendritic growth.

**Figure 3:**
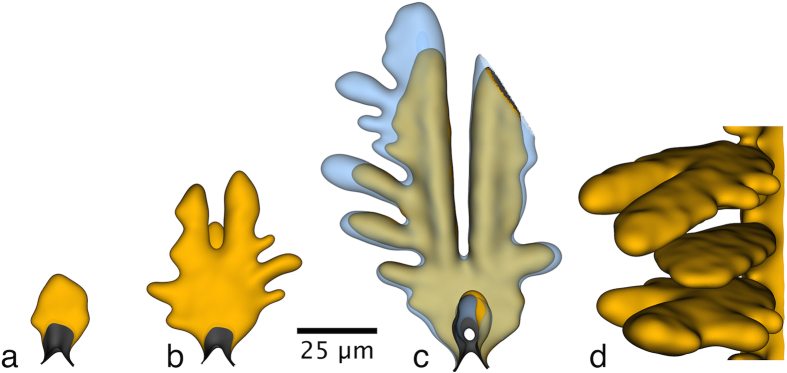
Evolution of a split tip of a secondary dendrite arm. (**a**) and (**b**) are one dendrite arm at 7.2, and 9.0 seconds and show the evolution of a splitting event. (**c**) shows a different arm at 10.8 seconds in yellow and at 14.4 seconds in a semi-transparent blue; this shows the large groove depths that are achievable and that the groove is very slow to evolve. (**d**) is a view of multiple branches showing the unusual morphology of the secondary arms.

The secondary arms in transparent organic materials, which are commonly used as metal analogs, display approximately cylindrical secondary arms with a well-defined tip^8^. By contrast, the secondary arms found in the Al dendrites are clearly not cylindrical, as shown in Fig. 3a. This very flat, almost plate-like appearance of the secondary arms is likely a result of diffusional interactions between secondary arms. This is likely a result of the comparatively small spacing of the secondary arms along the stem that thereby leads to diffusional interactions and flatter interfaces.

It is common to see the tips of these flat secondary arms split into two tips. An example of the initial dendrite tip splitting event is shown Fig. 3b,c. Note the initial broad shape of the dendrite arm in Fig. 3b that divides into two narrower arms in Fig. 3c. This occurs when the regions of high curvature connecting these flatter top and bottom surfaces, the “edges” of the secondary arm, undergo a morphological instability leading to multiple tertiary side branches and split tips. Tip splitting of primary dendrite tips has been observed during solidification in thin samples using X-ray radiography^26^, but the origin appears to be different from what is seen here.

A different split-tip secondary dendrite arm is shown in Fig. 3c at two times. This shows the depth of the grooves between the separate tips and also the lack of evolution of the groove. These deep grooves do not evolve due to the solute trapped inside them, and hence lead to regions of large segregation in the solid that exceeds that trapped between the secondary arms. Neither these very flat nor split tip secondary arms are seen in organic analogs^8^; thus the experiments demonstrate an important difference between model materials and metallic dendrites.

**Figure 4:**
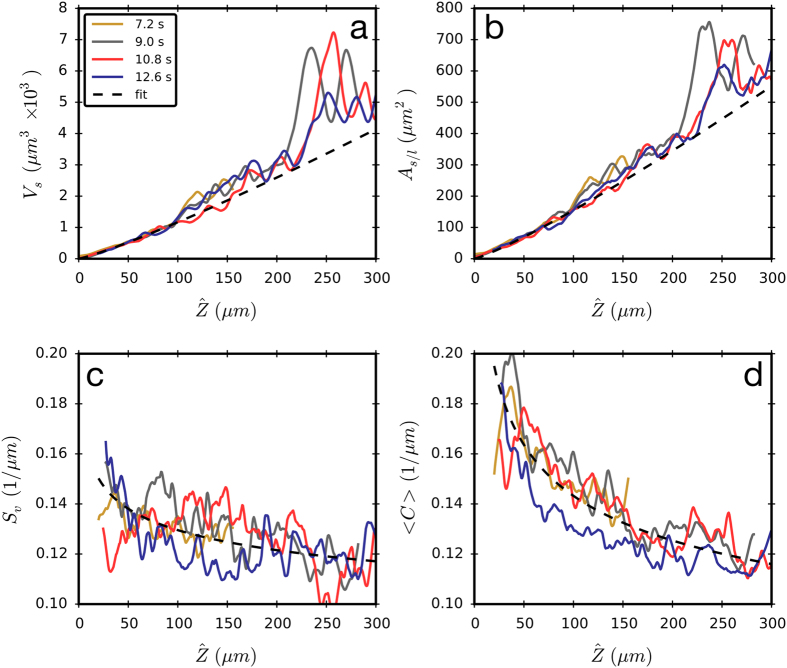
Properties as a function of distance from the dendrite tip. The (**a**) solid volume, (**b**) interfacial area, (**c**) surface area per volume, and (**d**) average curvature, as a function of position from the tip of the dendrite shown in Fig. 2a. The large variations in *V*_*s*_ and *A*_*s/l*_ are due to the development of large secondary branches. The tip radius, *R*, is approximately 1.5 μm, and thus *Z*/*R* is measured up to 200.

Dendrite morphology has been described theoretically and experimentally using transparent organic materials by determining the envelope of the tips of the secondary dendrite arms as a function of distance from the main tip of the dendrite^14^,^27^,^28^. These methods are extended here by using 3D data to calculate the total volume of solid (*V*_*s*_), solid/liquid interfacial area (*A*_*s/l*_), and the curvature (*C*) as a function of distance from the tip. The maximum distance sampled is considerable, approximately 200 tip radii. These are plotted in Fig. 4, along with the surface area per unit volume of solid, *S*_*v*_, the inverse of which is an analog to the average feature size^29^. The large fluctuations in *V*_*s*_ and *A*_*s/l*_ are associated with large secondary dendrite arms that are far from the tip, see the red region in Fig. 2.

Figure 4a,b show a dramatic increase in the volume of the dendrite and interfacial area with position back from the tip. However, when the ratio of these two quantities is computed there is only a slight decrease in *S*_*v*_, see Fig. 4c. This small increase in length scale is also evident in the decrease in the average curvature, 〈*C*〉, and shows that the increase in average feature size of the growing secondary arms is nearly balanced by the formation of smaller tertiary arms. Power laws are fitted to the volume and interfacial area data, using only the data within 0 < *Z*<100 *μm* to isolate the behavior of the primary arm and the growing secondary arms; this yields: *V*_*s*_ ∝ 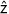
^1.16^, *A*_*s/l*_ ∝ 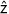
^1.15^. For the *S*_*v*_ and 〈*C*〉 data, the full range of data (0 < *Z* < 300μ*m*) is used in order to show how coarsening and the onset of tertiary arm formation affects the average size scale of the dendrite; the resulting fits are: *S*_*v*_ ∝ 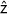
^−0.10^, and 〈*C*〉 ∝ 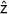
^−0.19^. By comparison, transparent organic analogue materials in the regime where only primary and secondary arms are present yield power laws of *V*_*s*_ ∝ 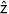
^2.10^, *A*_*s/l*_ ∝ 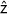
^1.92^, and *S*_*v*_ ∝ 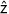
^−0.18^.^28^ The differences in the exponents show that the growth behavior of transparent organic analogs is quantitatively quite different from that of metals.

## Conclusions

We have examined the evolution of the complex morphology of the secondary dendrite arm structure of a dendrite growing into a supersaturated liquid. The morphology of the dendrite has been quantified in a manner appropriate for comparison to future simulations. We find a lack of self-similarity of the structure, no significant coarsening of the structure, power laws for the volume fraction transformed, surface area and surface area per volume that are different from those seen in transparent organic materials, and a markedly different side branch structure from that seen in transparent organic materials. The presence of tip splitting of the secondary arms implies that solute segregation should be present at levels that exceed those given by estimates of the secondary arm spacing. We expect that through improvements in camera frame rates and TIMBIR algorithms, still greater temporal resolution is possible in the future, thus making X-ray tomography the method of choice in studying dendritic growth in technologically important materials.

## Methods

### Time-interlaced, model-based, iterative reconstruction

The conventional approach to four-dimensional (4D) micro-tomography is to acquire a sequence of projection images of the object at progressively increasing view angles. Then, the projections for each half-rotation (π radians) are grouped together and reconstructed into a single 3D volume using an analytical reconstruction algorithm such as filtered back projection (FBP)^30^ or a Fourier domain reconstruction method^31^,^32^. The time sequence of 3D reconstructions then forms the 4D reconstruction of the object. For analytical reconstruction algorithms, the number of views required for Nyquist sampling of each 3D reconstruction is approximately the number of sampled pixels in the sensor’s cross-axial field of view. In practice, the large number of views required for each 3D reconstruction can dramatically reduce the temporal frame rate of the reconstructed 4D object. While the temporal frame rate can be increased by reducing the number of views per rotation, this results in under-sampling of the signal and produces substantial artifacts in the analytic reconstruction.

**Figure 5:**
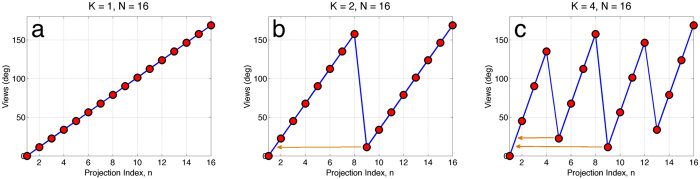
Illustration of interlaced view sampling pattern shown for (**a**) conventional progressive views or *K* = *1*, (**b**) interlaced views for *K* = *2*, and (**c**) interlaced views for *K* = *4*. The arrows point to the relative angular difference between two sub-frames.

To achieve high temporal resolution in 4D micro-tomography scans, we use the time-interlaced model-based iterative reconstruction (TIMBIR) method^20^. TIMBIR is a synergistic combination of two innovations: interlaced view sampling and model-based iterative reconstruction. The first innovation, interlaced view sampling, is a novel approach to data acquisition that distributes the needed view angles more evenly in time while limiting the required rotation speed of the sample (see Fig. 5). The second innovation, MBIR, computes the 4D reconstruction by searching for the result that best fits the sensor model (i.e., forward model) and image model (i.e., prior model)^19^,^33^. This allows TIMBIR to achieve reconstruction frame rates that are up to 16 times the conventional rate in our experiments. We expect that this technique will be useful in a broad range of time-dependent X-ray tomography experiments.

The first innovation of TIMBIR is interlaced view sampling, in which each *frame* of data, consisting of *N*_*θ*_ distinct views, is acquired over *K* interlaced *sub-frames*. Each *sub-frame* of data then consists of *N*_*θ*_/*K* equally spaced views, but together the full *frame* of data contains all *N*_*θ*_ distinct views of the object. The formula describing the view angle as a function of the discrete sample number, *n*, is given by:


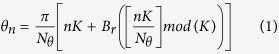


where *B*_*r*_(*a*) is the bit-reverse function which takes the binary representation of the integer, *a*, and reverses the order of the bits^34^. In interlaced view sampling, the views acquired over half a rotation using progressive view sampling, are instead acquired over multiple half-rotations. Fig. 5 compares progressive views with interlaced views and also highlights the interlacing of view angles across *sub-frames*.

Interlaced view sampling is a general technique and can be implemented in any X-ray imaging facility. In synchrotron imaging, the object is rotated at a constant speed and repeatedly imaged at the appropriate angles. In all our experiments, we are limited by the frame rate at which the camera can save images, *F*_*c*_. Thus, to implement interlaced view sampling, the detector is programmed to acquire images at the appropriate angles *θ*_*n*_ and the object is rotated independently at a sufficiently high speed such that the detector acquires images at the maximum frame rate. Consequently, since the number of views in *π* radian rotation of the object is lower in interlaced view sampling, the object is rotated at a higher speed than in progressive view sampling.

The second novelty in TIMBIR is to use a model-based iterative reconstruction (MBIR) algorithm, which is based on the estimation of a reconstruction, which best fits models of both the sensor measurements (i.e., the forward model) and the object (i.e., prior model). It is based on regularized inversion of the data typically with the form:


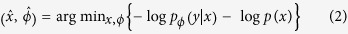


where *p*_*ϕ*_(*y*|*x*) is the forward model of the sensor measurements, *y*, given the reconstruction, *x*, and the unknown system parameters*, ϕ*, and *p*(*x*) is a prior model of the object in space and time. These models have been successfully used for volumetric reconstruction of 3D objects in, for example, medical applications^33^,^35^,^36^ in which they have been shown to substantially reduce X-ray dosage in medical CT scans^37^,^38^,^39^. For synchrotron imaging, *x* represents the 4D object; so the prior model can be used to regularize the solution in time as well as space. One important advantage of MBIR is that it allows for arbitrary sampling of the reconstruction in space and time. As it is used here, the projections in one sub-frame correspond to one time frame of the reconstruction and the prior model regularizes the reconstruction in space and across time frames. The forward model includes parameters that are part of *ϕ* that account for sensor noise, gain, and the anomalous measurements that typically result from sensor saturation due to high-energy particles^40^,^41^. These anomalous measurements are often referred to as “zingers” in synchrotron imaging^19^. The prior model is based on the use of a space-time Markov random field model (MRF) that is designed for independent control of space and temporal resolution. The actual form of the forward model and the prior model will be published in a future paper.

In Refs.[42,43], temporal regularization is used to improve reconstruction quality with progressive view sampling. However, in TIMBIR, temporal regularization effectively uses the interlacing of views across *sub-frames* to result in a synergistic improvement in reconstruction quality. With progressive views, the object is reconstructed once every *frame* at a rate of *F*_*s*_ = *F*_*c*_/*N*_*θ*_. Thus, to improve temporal resolution we can reduce the number of distinct views, *N*_*θ*_. However, this results in an under sampling of views which reduces the reconstruction quality and causes substantial artifacts^36^. Alternatively, with interlaced views, the object is reconstructed once every *sub-frame* at a rate of *F*_*s*_ = *KF*_*c*_/*N*_*θ*_. In this case, rather than reducing *N*_*θ*_, we instead increase *K* to improve the temporal resolution. Thus, the underlying idea of TIMBIR is to increase the number of interlaced *sub-frames* rather than reduce the number of distinct view angles.

### Sample preparation

The materials used here are prepared from 99.99% pure elements by the Materials Preparation Center at Ames Laboratory. The base materials are vacuum induction melted and cast into half-inch diameter rods, from which 1 mm diameter samples are cut using electrical discharge machining.

### Beamline setup

Experiments were run at the 2-BM beamline at Argonne National Laboratory’s Advanced Photon Source. Data is collected using polychromatic X-ray radiation to provide maximum X-ray flux. Imaging is done with a 100 μm thick LuAG:Ce scintillator and a PCO Edge CMOS camera that is equipped with a 10× magnifying objective to provide pixel sizes of 0.65 × 0.65 *μm*^2^. Images are cropped to 2048 × 1000 pixels, resulting in a bandwidth-limited maximum frame rate of approximately 70 frames per second; this is the limiting factor in the temporal resolution of the experiments. This image size yields a reconstruction size of 2048 × 2048 × 1000 with a voxel size of 0.65 × 0.65 × 0.65 *μm*^3^.

### Experiment details

During the experiment, the samples are held in a 3 mm diameter Boron Nitride (BN) holder, although the liquid is actually contained by a thin oxide shell on the outside of the sample. Samples are heated to 650 °C and cooled at a rate of 2 °C per minute using a furnace that is supplied by the 2-BM beamline. Given the 1 mm diameter of the sample, this cooling rate gives a uniform temperature within the sample. The thermal Péclet number is approximately 10^−6^, which for the alloy concentration employed in the experiment implies that the dendritic growth is solute-diffusion controlled.

The interlaced view sampling technique has *K* = 16 *sub-frames* and *N*_*θ*_ = 2000 projections per *frame*. Data is collected for approximately 115 seconds at a frame-rate of 70 Hz, resulting in a total of 8000 images, 4 *frames*, and 64 *sub-frames* of data being collected. Since the sample is reconstructed once every *sub-frame*, the time between reconstructions is 1.8 seconds.

During acquisition, the sample is rotated continuously at a rate of 100 degrees per second. This rotational speed of the sample yields a centripetal acceleration at the circumference of the sample that is about three orders magnitude smaller than gravitational acceleration; therefore, it can be assumed that the rotation does not affect the solidification process. Since the sample is rotating during the acquisition time of each projection, there will be some blurring due to sample rotation; however, the exposure time of the camera was set to 4 ms, which results in an almost negligible angular blur of 0.4 degrees during each sampling.

### Segmentation

Segmentation of this data and the isolated dendrite were done using a 3D, three-phase version of the method presented in^44^. This method results in a signed distance function to represent the interfaces, which can be used to calculate the interfacial curvatures using the method presented in^45^.

## Additional Information

**How to cite this article**: Gibbs, J. W. *et al*. The Three-Dimensional Morphology of Growing Dendrites. *Sci. Rep*. **5**, 11824; doi: 10.1038/srep11824 (2015).

## Supplementary Material

Supplementary Information
