# Supplementary material for: The Three-Dimensional Morphology of Growing Dendrites
Source: Sci Rep. 2015 Jul 3;5:11824. doi: 10.1038/srep11824 (PMC4490335; doi:10.1038/srep11824)
Supplement: Supplementary Information [file srep11824-s1.pdf]

Title: The Three-Dimensional Morphology of Growing Dendrites

Authors: J.W. Gibbs[1], K.A. Mohan[2], E.B. Gulsoy[1], A. Shahani[1], X.Xiao[3], C. Bouman[2], M. De Graef[4], P.W. Voorhees[1]*

Affiliations:

[1] Department of Materials Science and Engineering, Northwestern University, Evanston, IL

[2] Department of Electrical and Computer Engineering, Purdue University, West Lafayette, IN

[3] Argonne National Laboratory, Lemont, IL 60439

[4] Department of Materials Science and Engineering, Carnegie Mellon University, Pittsburgh, PA

* Correspondence to: p-voorhees@northwestern.edu

Supplementary information:

Comparison of reconstruction methods

To assess and demonstrate the utility of the time-interlaced model-based iterative reconstruction (TIMBIR) method, reconstructions of a phantom are done using TIMBIR and several alternative methods. In a real physical system, since the voxel values of the object vary with time, every projection is of a different 3D object. Thus, to accurately model the data acquisition in a physical system, simulated data is generated using a time and space varying phantom of a two-phase material system with attenuation coefficients of 2.0mm^{-1} and 0.67mm^{-1}. The phantom is generated using the Cahn-Hilliard equation, which models the process of phase separation in the cross-axial plane (*x-y* axes). The 2D phantom is then repeated along the axial dimension (*z*-axis), to get a time-varying phantom in 3D space.

The phantom is assumed to have a voxel resolution of $0.65 \times 0.65 \times 0.65 \mu m^3$ and a size of $N_x \times N_y = 1024 \times 1024$ in the cross-axial plane. To generate projections, the phantom is then sampled in time at the data collection rate, F_c, and forward projected at the appropriate angles. The reconstructions in Fig. S1 are of simulated datasets with $N_\theta = 256$ distinct angles per *frame,* with $K = 16$ *sub-frames* (interlaced views) and $K = 1$ *sub-frame* (progressive views). The simulated sensor has a resolution of 256 pixels in the cross-axial direction and 4 pixels in the axial direction. Each reconstructed slice has a voxel resolution of $2.6 \times 2.6 \times 2.6 \mu m^3$ and a size of $N_x \times N_y = 256 \times 256$. The 4D reconstruction has one 3D volume in every *sub-frame,* which results in a reconstruction frame rate of $F_c K / N_\theta$, where F_c is the frame rate of the camera. Since, the reconstruction frame rates are different for different values of K, to facilitate comparison, the reconstructions are up-sampled by interpolating to the camera frame rate, F_c. Also, the phantom is down-sampled in 3D space to the reconstruction resolution before comparison. In Fig. S1, we show a cross-axial slice of a 3D volume of the up sampled 4D reconstruction using different methods.

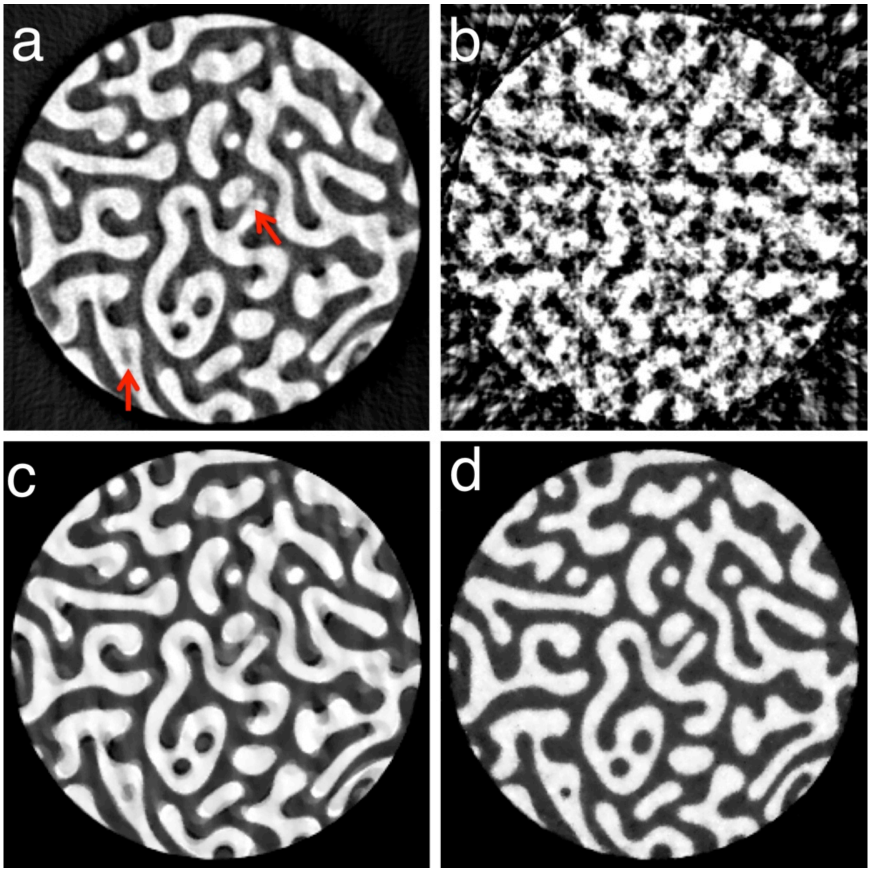

Fig. S1. A cross-axial slice of a 3D volume from the 4D reconstruction of simulated data. Reconstructions show (a) FBP with progressive scanning, (b) FBP/interlaced, (c) MBIR/progressive and (d) MBIR/interlaced. The TIMBIR result synergistically combines MBIR with interlaced view sampling to increase temporal resolution by a factor of approximately 16, while maintaining spatial resolution. The arrows point to areas where TIMBIR performs significantly better than the conventional method.

Table S1. Root mean squared error between the reconstructions and the phantom ground-truth.

**Method**	**RMSE** (mm^{-1})
FBP/progressive	0.2528
FBP/interlaced	0.5867
MBIR/progressive	0.1032
TIMBIR	0.0853

Figure S1 shows simulation results that illustrate the power of TIMBIR in increasing both temporal and spatial resolution. The main artifacts in the standard method of FBP with

progressive sampling are the blur rings and distortions. Notice that interlaced view sampling works poorly when combined with traditional FPB reconstruction. And while MBIR reconstruction can improve quality, it still suffers from artifacts when used with traditional progressive sampling. However, the combination of interlaced view sampling and MBIR results in a synergistic image quality improvement that forms the core benefit of the TIMBIR method. The root mean squared error (RMSE) between the reconstructions and the known phantom ground-truth (see Table S1) support these visual conclusions.
